# Dynamic effects of microglial NLRP3 on transcriptional alterations post-traumatic brain injury

**DOI:** 10.3389/fnins.2026.1951885

**Published:** 2026-09-23

**Authors:** Ruo-Bing Gao, Chang-Hong Li, Nan Yang, Xing Chen, Bo Wang, Dan Xu, Gan Luo, Zi-Jian Yan, Ya-Lei Ning, Hao Du, Yong-Gang Liu

**Affiliations:** 1 Laboratory of Stem Cell and Tissue Engineering, Chongqing Medical University, Chongqing, China; 2 The Molecular Biology Center, State Key Laboratory of Trauma and Chemical Poisoning, Department of Army Occupational Disease, Da-ping Hospital, Army Medical University (Third Military Medical University), Chongqing, China; 3 Department of Anatomy, Anhui Medical University, Hefei, Anhui, China; 4 Department of Neurosurgery, The First Affiliated Hospital of Chongqing Medical University, Chongqing, China; 5 Department of Urology, General Hospital of Xizang Military Command, Lhasa, China

**Keywords:** CNS, microglia, NLRP3, transcriptome, traumatic brain injury

## Abstract

**Introduction:**

The nucleotide-binding oligomerization domain, leucine-rich repeat, and pyrin domain-containing protein 3 (NLRP3) inflammasome is predominantly expressed in microglia within the central nervous system (CNS) and plays critical roles in neuroinflammatory diseases, including traumatic brain injury (TBI). The function of the microglial NLRP3 inflammasome may vary as the disease progresses. A comprehensive understanding of these dynamic alterations is essential for opti-mizing the therapeutic time window. Post-TBI, microglial NLRP3 levels exhib-ited significant elevation, and microglial-specific NLRP3 conditional knockout (NLRP3-CKO) reduced neurological and neuropathological impairments.

**Methods:**

Transcriptome sequencing was performed at baseline and on days 1, 3, and 7 post-TBI using injured cortical tissue from NLRP3-CKO mice and their litter-mate controls.

**Results:**

Notable transcriptional changes occurred on day 3 post-TBI, while fewer differentially expressed genes (DEGs) were identified at other time points. NLRP3-CKO influenced macrophage polarization on day 1 and neuronal cell survival on day 3 post-injury. In addition, NLRP3-CKO significantly reduced the upregulation of pyroptosis-related genes and the dysregulation of genes associ-ated with neuroinflammation and neuropathology after TBI. Gene set enrichment analysis (GSEA) indicated diverse potential regulatory pathways at various time points. The temporal profiles of gene expression were also analyzed, revealing distinct expression trends in several gene clusters, particularly on day 7 post-TBI. Pathway enrichment analysis of overlapping genes in these clusters identified several relevant pathways, which were further validated through quantitative real-time polymerase chain reaction (qPCR).

**Discussion:**

Overall, these findings elucidate the dynamic effects of microglial NLRP3 on transcriptomic alterations during the acute phase of TBI and highlight potential regulatory pathways, offering insights into NLRP3 inflammasome-related pathological mechanisms and enhancing the optimization of therapeutic time windows for TBI.

## 1 Introduction

The nucleotide-binding oligomerization domain, leucine-rich repeat, and pyrin domain-containing protein 3 (NLRP3) inflammasome is a vital component of the innate immune system, regulating host immune homeostasis. Numerous pathogen-associated molecular patterns (PAMPs) and damage-associated molecular patterns (DAMPs) can activate NLRP3 signaling cascades, resulting in the release of inflammatory cytokines (IL-1β and IL-18) and pyroptosis (de Rivero Vaccari et al., 2026). In the central nervous system (CNS), microglia primarily drive NLRP3 inflammasome signaling, with its activation playing pivotal roles in various neuroinflammation-related diseases, including traumatic brain injury (TBI), Parkinson’s disease, Alzheimer’s disease, and stroke (Kodi et al., 2026). Researchers increasingly focus on identifying therapeutic targets through the inhibition or blockade of NLRP3 inflammasome activation (Yao et al., 2024). Several chemical compounds have demonstrated potential neuroprotective effects against NLRP3 signaling-related targets, including NLRP3 itself and caspase-1 (Guo et al., 2026; Oguzoglu et al., 2026; Smidt et al., 2026). However, NLRP3 inflammasome activation exhibits a dual nature, with its roles in disease varying over time. In an ischemia–reperfusion injury model, inhibiting NLRP3 signaling did not reduce infarct size at early time points (1–3 h), with benefits emerging only 6–24 h after reperfusion (Zuurbier, 2019). Thus, further elucidation of the effects of NLRP3 inflammasome inhibition or deficiency across different time windows in CNS diseases is warranted.

More than 50 million individuals experience TBI annually, and half of the global population is expected to encounter one or more incidents over their lifetime (Jiang et al., 2019). Consequently, TBI represents a significant public health challenge. Activation of the NLRP3 inflammasome and the resultant neuroinflammation play critical roles in secondary injury after TBI (Chakraborty et al., 2023). Numerous studies have shown that NLRP3 inhibition or deficiency confers neuroprotective effects post-TBI (Miao et al., 2025; Cai et al., 2026). Nevertheless, anti-inflammatory therapies have not demonstrated clinical benefits, potentially due to the protective effects of neuroinflammation at certain stages post-TBI (Simon et al., 2017). For instance, tumor necrosis factor (TNF) deficiency—an upstream signaling molecule involved in NLRP3 activation—bestows early neuroprotection but results in impaired motor function and larger lesions four weeks post-TBI (Simon et al., 2017). Thus, investigating the dynamic effects of NLRP3 inflammasome signaling in the context of TBI is crucial for understanding its differential roles and for selecting appropriate therapeutic windows for NLRP3-targeted therapies.

This study investigated the dynamic effects of NLRP3 conditional knockout (NLRP3-CKO) on transcriptomic changes during the acute phase after TBI. The results revealed time-dependent alterations in immune and central nervous system responses, further elucidating the underlying mechanisms. This study enhances our understanding of the dynamic roles of the NLRP3 inflammasome in TBI and informs the application of NLRP3-targeted drugs for treating secondary injury following TBI.

## 2 Methods

### 2.1 Animals

Adult male NLRP3-CKO mice and their littermate CX3CR1^CRE/ERT2^ (CRE) mice on a C57BL/6 J genetic background (aged 10–12 weeks) were utilized in this study. NLRP3-CKO mice were generated as previously reported (Du et al., 2024). Briefly, NLRP3^flox/−^ mice were crossed with CRE mice to generate NLRP3^flox/flox^/CX3CR1C^RE/ERT2+^ mice (hereafter referred to as NLRP3^CX3CR1^ mice). To activate Cre recombinase, five consecutive daily intraperitoneal injections of tamoxifen (75 mg/kg, 20 mg/mL in corn oil; Sigma) were administered. Both NLRP3^CX3CR1^ and CRE mice received tamoxifen treatment to eliminate potential off-target effects of Cre recombinase. One month after tamoxifen administration, successful NLRP3 knockout was confirmed in NLRP3^CX3CR1^ mice, and all tamoxifen-treated mice were subsequently used for experiments. Genotyping of transgenic mice was conducted through agarose gel electrophoresis, with gene amplification primers listed in Supplementary Table S1.

Mice were housed in the animal center of Daping Hospital, Army Medical University (Facility Certificate No. SCXK [Yu] 2002–0002; Chongqing, China). All animal experiments obtained approval from the Laboratory Animal Welfare and Ethics Committee of Army Medical University (Approval NO. AMUWEC20191822) and adhered to the ARRIVE guidelines and the NIH Guide for the Care and Use of Laboratory Animals.

### 2.2 TBI model

Tamoxifen-induced NLRP3-CKO and littermate CRE mice were subjected to a moderate TBI model using the controlled cortical impact method, as previously described (Zeng et al., 2018). Mice were anesthetized with 2.5% tribromoethanol (25 mg/kg), followed by a 5-mm-diameter craniotomy performed between the bregma and lambdoid sutures over the left parietal cortex. A controlled cortical impact was delivered using an aerodynamic impact device (PSI, USA) equipped with a 3-mm-diameter metal tip, impacting 2 mm below the dura at a velocity of 3.5 m/s. Sham-operated mice underwent the same surgical procedure as TBI mice, excluding the impact injury.

### 2.3 Western blot (WB)

Western blot (WB) analysis was conducted as previously reported (Du et al., 2024). In brief, injured cortical tissues were collected and lysed in RIPA buffer (Thermo Fisher), and proteins were denatured. Protein samples were separated by electrophoresis on 10% stain-free SDS-PAGE gels (Bio-Rad) and transferred onto polyvinylidene difluoride (PVDF) membranes. Total protein was visualized using Bio-Rad stain-free technology with a ChemiDoc imaging system (Bio-Rad). The PVDF membranes were then blocked with goat serum and incubated overnight at 4 °C with an anti-NLRP3 antibody (Adipogen, AG-20B-0014, 1:1000). Following overnight incubation, the membranes were washed five times with Tris-buffered saline containing Tween 20 (TBST) and incubated with a horseradish peroxidase-conjugated anti-mouse secondary antibody (Bioworld, BS12478, 1:10000) at room temperature for one hour. After additional washes, protein bands were visualized, with total protein serving as the loading control. All data were analyzed using the Image Lab software (Bio-Rad).

### 2.4 Immunofluorescence

Immunofluorescence was performed as previously described (Zeng et al., 2018). In brief, mouse brains were fixed in 4% paraformaldehyde (PFA) and sectioned into 30-μm-thick coronal cryosections using a microtome. The sections were washed with PBS and permeabilized with 0.3% Triton X-100, followed by blocking with 10% goat serum. The sections were then incubated overnight at 4 °C with primary antibody solutions (anti-Iba-1: Abcam, ab178846, 1:200 dilution; anti-NLRP3: Adipogen, AG-20B-0014, 1:100 dilution; anti-NeuN: Millipore, MAB-377, 1:100 dilution; anti-MAP2: Abcam, ab32454, 1:500). The following day, the sections were washed five times with PBST (PBS containing 0.1% Tween-20) and incubated with appropriate secondary antibodies at room temperature in the dark. Nuclear counterstaining was performed with DAPI (10 μg/mL, Sigma), and the sections were mounted with an antifade mounting medium.

### 2.5 Neurobehavioral tests

NLRP3-CKO mice were designated as the experimental group, while littermate CRE mice were assigned to the sham and TBI groups. Previous studies have confirmed that NLRP3-CKO does not affect baseline neurological functions in mice prior to TBI (Zeng et al., 2018). Neurobehavioral assessments were conducted at baseline and on days 1, 3, and 7 post-TBI. Mice were randomly assigned to each group, with all tests carried out by experimenters blinded to the genotypes and treatments of the animals.

#### 2.5.1 Neurological severity score (NSS) test

The Neurological Severity Score (NSS) test, which evaluates neuromuscular, vestibulomotor, and complex neuromotor functions, was performed as previously detailed (Du et al., 2022). Scores ranged from 0 to 20, where 0 indicated normal function and 20 represented the most severe neurological deficits.

#### 2.5.2 Grip strength test

The grip strength test was conducted to evaluate neuromuscular function post-TBI (Shen et al., 2026). A 50-cm-long straight string was tensioned between two 1-m-tall vertical supports, and mice were positioned at the midpoint of the string. Scoring was performed based on the following criteria: 0: falls off the string; 1: hangs on with both forepaws; 2: similar to 1 but attempts to climb; 3: hangs on with forepaws and one or both hind paws; 4: holds on with all four paws, with tail wrapped around the string; and 5: escapes to one of the vertical supports.

#### 2.5.3 Accelerating rotarod test

The accelerating rotarod test, used to assess gross motor function and balance, was administered as previously described (Zeng et al., 2018). Mice were acclimatized to the rotarod at 4 rpm for three days prior to injury. During the test, mice were placed on a rod that accelerated from 4 to 40 rpm over 300 s. The latency to fall or passively cling to the rod was recorded, with three trials conducted per mouse and latency values averaged.

#### 2.5.4 Open field test

The open field test was conducted to evaluate spontaneous locomotor activity and anxiety-like behaviors, as previously reported (Miao et al., 2023). The test was performed in a 40 × 40 × 50 cm open field apparatus for a duration of 5 min, with the total distance traveled and time spent in the central zone (20 × 20 cm) recorded.

### 2.6 Cerebral water content measurement

Cerebral water content was measured using the wet-dry weight method at 24 h post-TBI, as previously described (Yan et al., 2022). At this time point, mice were anesthetized, and the injured parietal cortex (impact site) was rapidly dissected, weighed to obtain its wet weight, and placed on tinfoil. The samples were dried at 100 °C for one day and then reweighed. Cerebral water content was calculated using the following formula: cerebral water (%) = (wet weight – dry weight) / wet weight × 100.

### 2.7 Quantitative real-time PCR (qPCR)

Quantitative real-time polymerase chain reaction (qPCR) was conducted following previously established protocols (Du et al., 2024). Total RNA was extracted using an RNA isolation kit (Promega), with RNA purity and concentration assessed using a NanoDrop One UV–Vis spectrophotometer. Reverse transcription was performed using the GoScript™ Reverse Transcription Kit (Promega) according to the manufacturer’s instructions. Complementary DNA (cDNA) amplification was performed using GoTaq® qPCR Mix (Promega) and target-specific primers (Supplementary Table S1). The thermal cycling protocol included an initial denaturation step at 95 °C for 10 min, followed by 40 cycles of denaturation at 95 °C for 15 s and 60 °C for 1 min. Relative mRNA expression levels were quantified using the 2^−ΔΔCt^ method, employing housekeeping genes as endogenous references.

### 2.8 RNA sequencing

Parietal cortices from sham-operated or TBI-treated CRE or NLRP3-CKO mice (sham and days 1, 3, and 7 post-TBI; three mice per group per time point, totaling 24 mice) were dissected and rapidly flash-frozen in liquid nitrogen. These samples were subsequently processed for RNA sequencing by Shanghai Majorbio Biopharm Technology Company. Total RNA was extracted using TRIzol Reagent, with genomic DNA removed by DNase I. Only high-quality RNA samples (OD260/280 = 1.8–2.2, OD260/230 ≥ 2.0, RIN ≥ 6.5, 28S:18S ≥ 1.0, and > 1 μg per sample) were utilized for sequencing library construction. The TruSeq™ RNA Sample Prep Kit (Illumina, FC-122-1002) was employed following the manufacturer’s instructions. Paired-end RNA-seq libraries were sequenced on an Illumina HiSeq X Ten/NovaSeq 6,000 sequencer with a read length of 2 × 150 bp. Raw paired-end reads underwent trimming and quality control using SeqPrep^1^ and Sickle^2^ with default parameters. Clean reads were aligned to the reference genome (GRCm39) in orientation mode using the HISAT2 software (Kim et al., 2015). Mapped reads from each sample were assembled using StringTie with a reference-guided approach (Pertea et al., 2015). Transcript expression levels were calculated using the transcripts per million (TPM) metric, and RSEM was employed to quantify gene abundances (Li and Dewey, 2011). Transcriptome sequencing was performed on the 24 samples, with raw reads preprocessed to trim adapter sequences and filter low-quality reads, yielding clean reads for subsequent analyses. Sequencing quality assessments confirmed high standards, with a Q30 base percentage exceeding 94.8% for all samples. Clean reads from each sample were mapped to the specified reference genome, achieving mapping rates between 96.48 and 97.35%. Expression quantification yielded a total of 35,340 expressed genes (34,551 annotated genes and 789 novel genes) and 132,678 expressed transcripts (107,863 known transcripts and 24,815 novel transcripts) across all samples.

### 2.9 Dimensionality reduction, hierarchical clustering, and differentially expressed gene (DEG) analysis

Principal component analysis (PCA) and t-distributed Stochastic Neighbor Embedding (t-SNE) were used to facilitate dimensionality reduction analysis. The “prcomp” function from the R “stats” package was employed for PCA, while the “Rtsne” package was utilized for t-SNE analysis.

Unsupervised hierarchical clustering based on the Ward. D method was conducted using the “hclust” function from the R “stats” package with a Euclidean distance matrix.

Differential gene expression analysis between NLRP3-CKO and CRE groups was performed at each time point (sham, 1, 3, and 7 days post-TBI) using the DESeq2 R package. Genes exhibiting an adjusted *p*-value of < 0.05 and an absolute fold change of > 1.4 were classified as differentially expressed genes (DEGs) (Love et al., 2014). Volcano plots visualizing DEG distributions were generated using the EnhancedVolcano R package.^3^ For temporal dynamics analysis, log_2_ fold change values (relative to corresponding sham controls) were calculated separately for the NLRP3-CKO TBI and CRE TBI groups utilizing DESeq2. Fold change values were subsequently compared between genotypes across the three post-TBI time points.

### 2.10 Cell composition analysis

Gene set variation analysis (GSVA) was performed using the GSVA R package (Hänzelmann et al., 2013) based on cell-type signature gene sets (M8) derived from the Mouse MSigDB.^4^ The M8 gene sets comprise curated cluster markers for cell types identified in single-cell sequencing studies of mouse tissues.

Immune cell composition estimation was performed using the CIBERSORT R package (Newman et al., 2015) based on a published mouse immune cell gene signature matrix (Chen et al., 2017).

In addition, a deconvolution method was employed to analyze neural cell composition (Lu et al., 2021), encompassing the following cell groups: Neurons, astrocytes, glial cells, oligodendrocytes, ependymal cells, and fibroblasts.

### 2.11 Dynamic changes in pyroptosis-, neuroinflammation-, and neuropathology-related gene analysis

Pyroptosis-related genes (83 genes) were sourced from PubMed using a gene retrieval and prioritization tool (GLAD4U) (Jourquin et al., 2012). Neuroinflammation-related (745 genes) and neuropathology-related (750 genes) gene sets were downloaded as previously described (Du et al., 2024). TPM values for these genes were normalized using the z-score method and visualized. Neuroinflammation- and neuropathology-related genes were categorized into upregulated and downregulated groups based on expression changes on day 3 in the CRE group versus the CRE sham group, and the gene sets were analyzed separately.

Over-representation analysis (ORA) based on KEGG and WikiPathways pathway annotations for pyroptosis-, neuroinflammation-, and neuropathology-related genes was carried out using the clusterProfiler R package (Xu L. et al., 2024; Xu S. et al., 2024).

### 2.12 Gene set enrichment analysis (GSEA)

Gene set enrichment analysis (GSEA) utilizing mouse ortholog Hallmark gene sets from MSigDB, WikiPathways, and KEGG pathway gene sets was conducted using the clusterProfiler R package (Xu L. et al., 2024; Xu S. et al., 2024). The expression matrix was first analyzed using the DESeq2 package, and genes were ranked by log_2_ fold change prior to GSEA.

### 2.13 Temporal clustering and pathway enrichment

A soft clustering approach based on the fuzzy c-means (FCM) algorithm (Kumar and Futschik, 2007), implemented in the Mfuzz R package, was used to identify temporal gene expression clustering. This analysis was performed separately for the CRE and NLRP3-CKO groups, identifying six clusters for each group. Subsequent KEGG pathway enrichment analysis was conducted on the genes within each cluster.

Pairwise gene intersections between the corresponding clusters of the two groups were calculated, and genes from the four largest intersections were selected for KEGG pathway enrichment analysis.

### 2.14 Statistical analysis

Data are presented as mean ± standard deviation (SD). Behavioral data were evaluated for normality using the Kolmogorov–Smirnov test (*p* > 0.1) and analyzed via two-way ANOVA followed by Tukey’s multiple comparisons test. Quantitative real-time PCR data analysis was performed using the unpaired two-tailed Student’s *t*-test. Behavioral statistical analyses were conducted using the GraphPad Prism 8 software. Expression analyses of pyroptosis-, neuroinflammation-, and neuropathology-related genes were performed using a two-factor non-parametric test (Scheirer–Ray–Hare test), followed by Dunn’s test for *post hoc* pairwise comparisons, using the rstatix R package. For comparisons involving three groups, one-way ANOVA followed by Tukey’s multiple comparisons test was applied. A *p*-value of < 0.05 was considered statistically significant.

## 3 Results

### 3.1 Microglial NLRP3 was elevated post-TBI, and NLRP3-CKO attenuated neurological deficits and neuropathological alterations post-TBI

The expression of NLRP3 and the effects of NLRP3-CKO on TBI were assessed. Our results demonstrated a significant elevation in NLRP3 levels post-TBI, with prominent co-localization observed with Iba-1^+^ cells (Figures 1A–C). NLRP3-CKO mice were generated through tamoxifen induction, and genotypes were confirmed via agarose gel electrophoresis (Figures 1D,E). Neurobehavioral tests were conducted to evaluate the neurological deficits in NLRP3-CKO mice following TBI. The NLRP3-CKO group exhibited lower NSS scores and superior performance in the grip strength and accelerating rotarod tests compared to the TBI group, particularly on days 3 and 7 post-injury (Figures 2A–C). In the open field test, mice in the NLRP3-CKO group traveled a longer total distance than those in the TBI group on days 3 and 7 post-TBI, with NLRP3-CKO mice spending more time in the central zone on day 7 post-injury (Figures 2D,E). These observations indicate that NLRP3-CKO provides protective effects against deficits in spontaneous locomotor activity and anxiety-like behaviors post-TBI. Furthermore, NLRP3-CKO reduced cerebral edema at 24 h post-TBI and increased the intensity of MAP2^+^ cells on day 7 post-injury while exhibiting no significant effects on NeuN^+^ cells (Figures 2F–I). These findings suggest that microglial NLRP3 plays a critical role in secondary injury following TBI and that NLRP3-CKO exerts significant neuroprotective effects post-injury.

**Figure 1:**
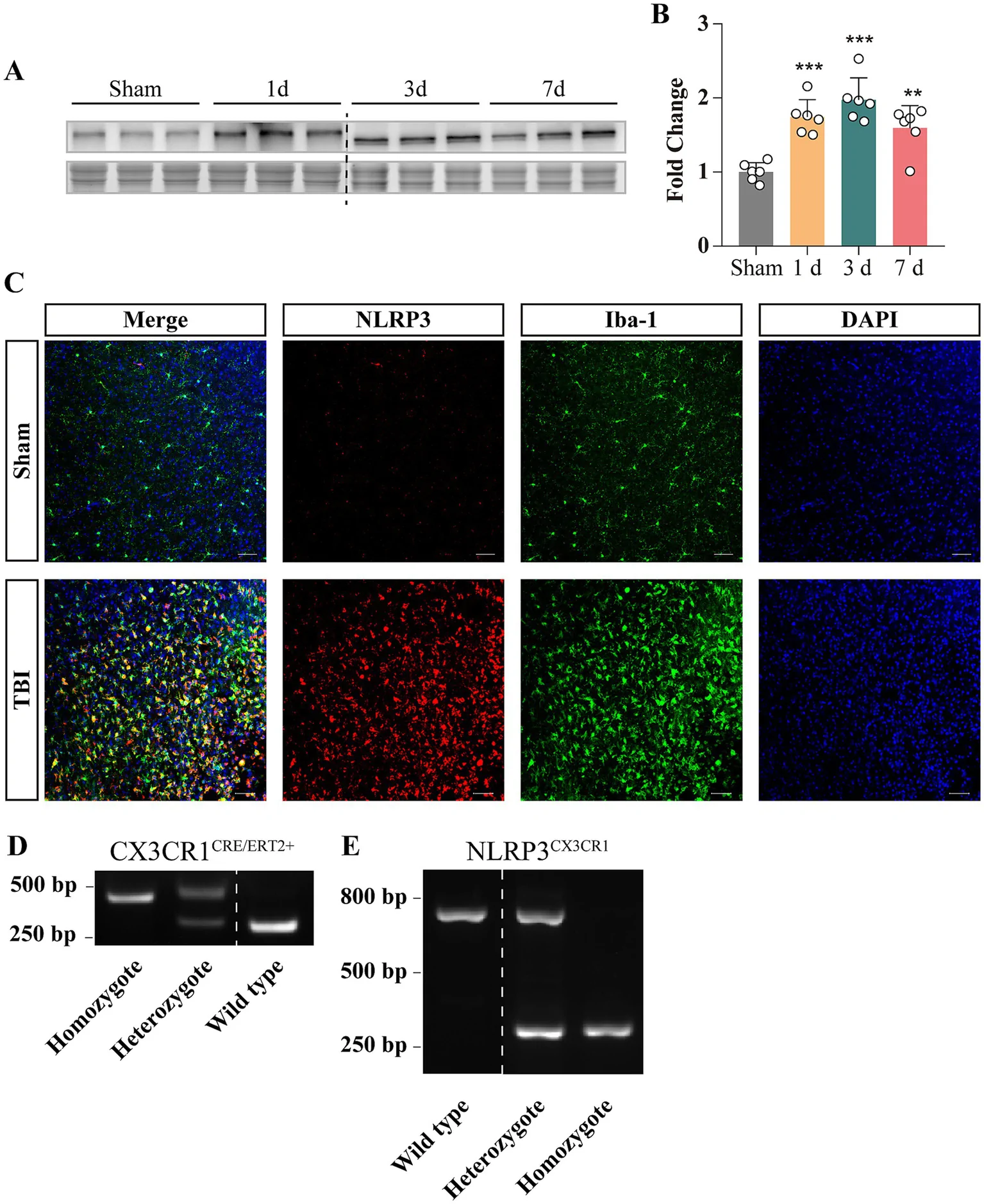
Microglial NLRP3 was significantly elevated post-TBI. **(A,B)** Representative immunoblot images and quantitative analysis of NLRP3 expression at 1 d, 3 d, and 7 d post-TBI (one-way ANOVA followed by Tukey’s multiple comparisons test, *p < 0.05, *n* = 6). **(C)** Representative immunofluorescence images of NLRP3 (red) and Iba-1 (green). DAPI was used for nuclear staining. Scale bar = 50 μm. **(D,E)** Representative agarose gel electrophoresis images showing genotyping results for CRE and NLRP3-CKO mice.

**Figure 2:**
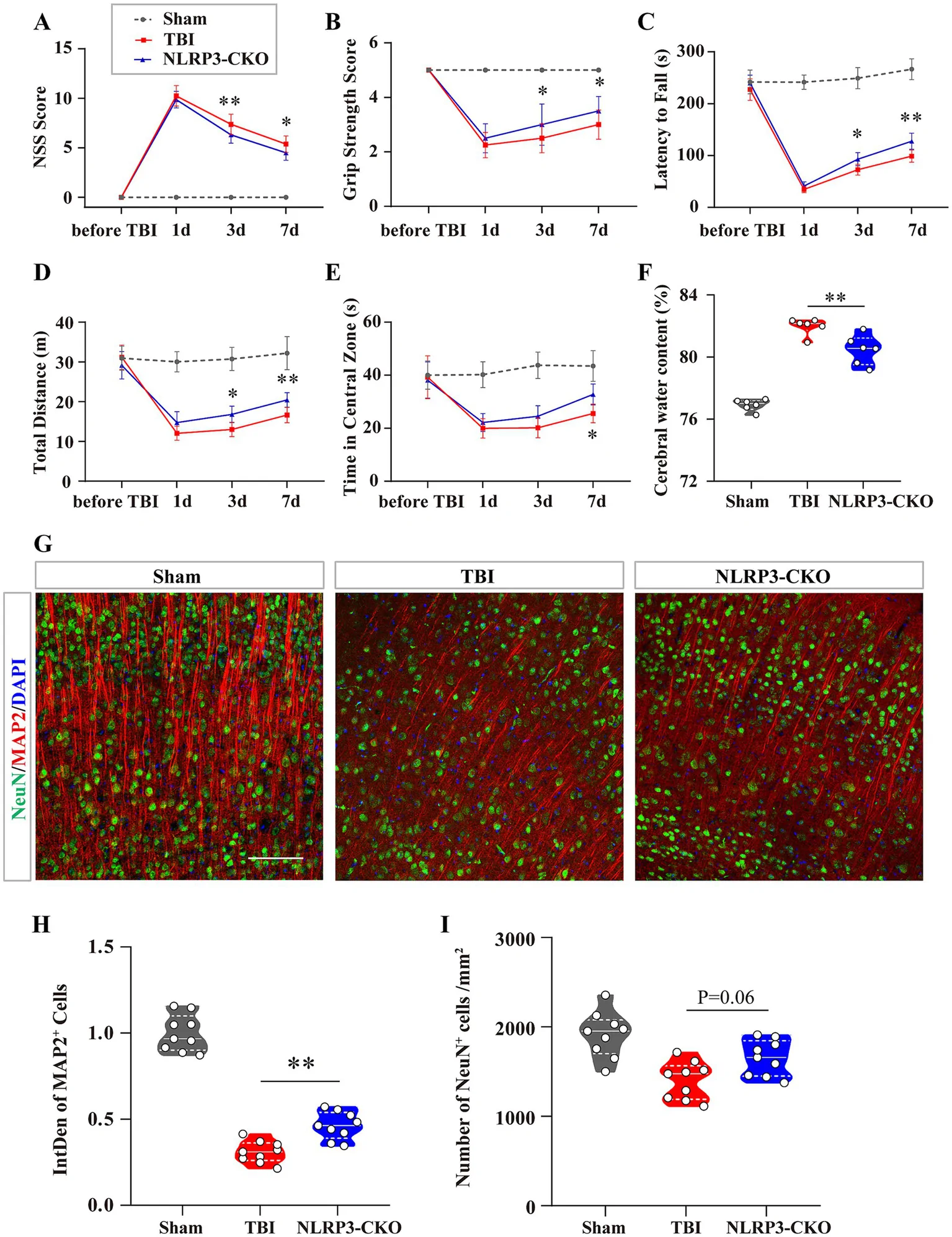
Microglial NLRP3-CKO attenuated neurological deficits and neuropathological alterations post-TBI. **(A–C)** Results of the NSS, grip strength, and rotarod tests at different time points across all groups (*p < 0.05, **p < 0.01). **(D,E)** Quantitative analysis of the total travel distance (cm) and time spent in the central zone (s) in the open field test at distinct time points across all groups. **(F)** Cerebral water content of mice in different groups (*n* = 3). Behavioral data passed the Kolmogorov–Smirnov normality test (**p < 0.01) and were then analyzed using two-way repeated measures ANOVA followed by Tukey’s multiple comparisons test. **(G)** Representative immunofluorescence images of NeuN (neuronal nuclei, green), MAP2 (dendritic cytoskeleton, red), and DAPI (nuclear counterstain, blue), showing neuronal and dendritic structures in Sham, TBI, and NLRP3-CKO mice. Scale bar = 100 μm. **(H,I)** Quantitative analysis of the integrated intensity of MAP2-positive cells and the number of NeuN-positive cells (*n* = 9, *p < 0.05, **p < 0.01).

### 3.2 NLRP3-CKO caused striking transcriptional alterations, primarily on day 3 post-TBI

RNA sequencing was performed to explore the dynamic effects of NLRP3-CKO on transcriptional alterations post-TBI. PCA, t-SNE, and hierarchical clustering analyses were performed to illustrate the global sample distribution post-TBI. The results indicated that NLRP3-CKO samples were distinctly separated from CRE samples on day 3 post-injury (Figures 3A–C). DEG analyses between the NLRP3-CKO and CRE groups at various time points revealed striking transcriptional changes, primarily on day 3 post-TBI, with fewer DEGs observed on days 1 and 7 post-injury (Figures 3D–G). Moreover, comparisons of fold change values in gene expression between the NLRP3-CKO and CRE groups versus their corresponding sham groups on different post-injury days provided additional insights. NLRP3-CKO mice displayed globally lower levels of gene upregulation or downregulation across all time points, with several reversed expression patterns compared to CRE mice (day 1: down-to-up: Fkbp5; up-to-down: Sfrp5, Gmnc, Acss3; day 3: down-to-up: Syt6, Gm37421, 5330416C01Rik; up-to-down: Pagr1a, Krt18, Gm9531, Btg2, Bace2; day 7: up-to-down: Zfp91, Fat4). These findings illustrate that the transcriptional effects of NLRP3-CKO on TBI were most pronounced on day 3 post-injury, while also exhibiting fine-tuning effects at all evaluated time points.

**Figure 3:**
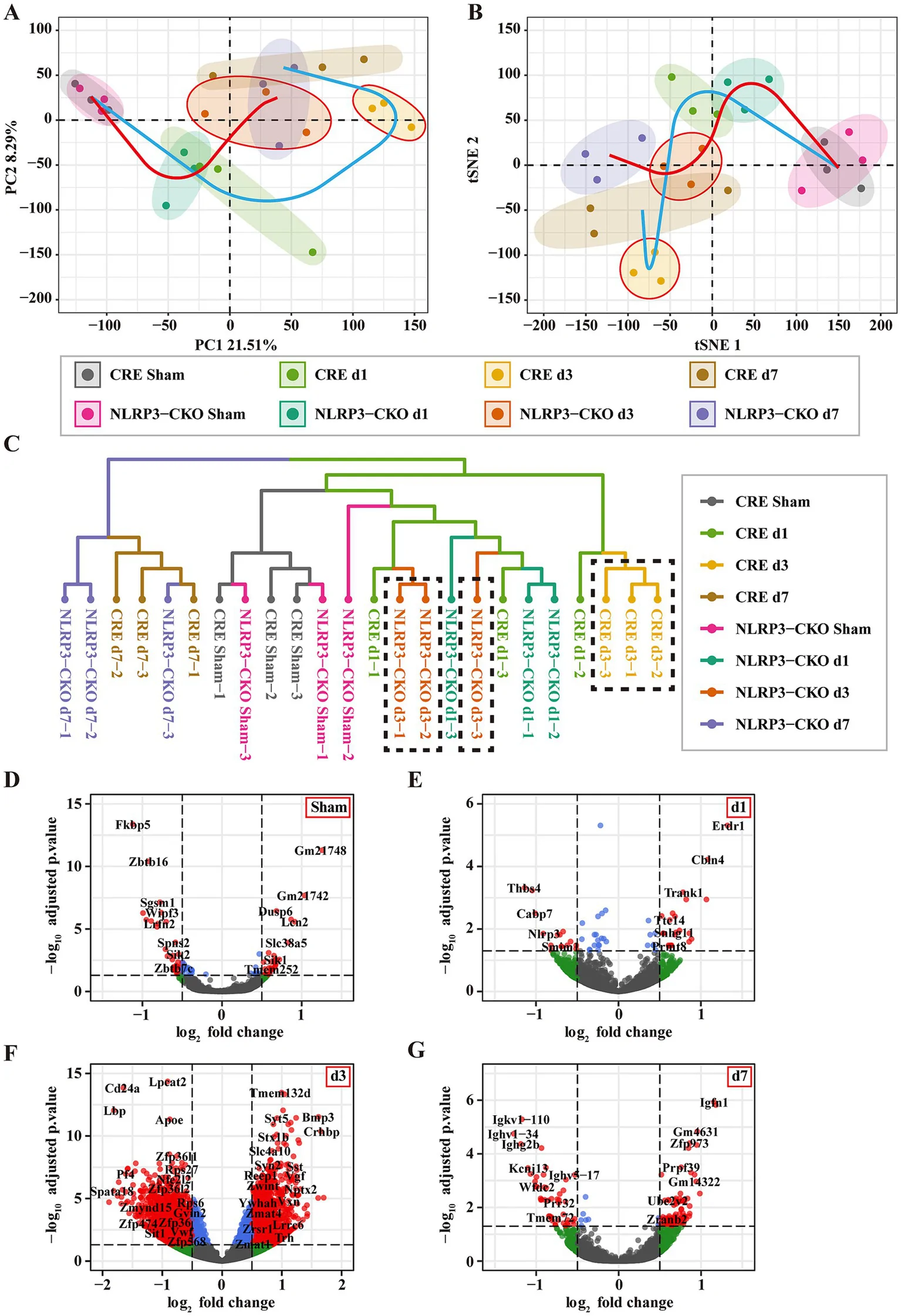
NLRP3-CKO induced prominent transcriptional alterations, primarily at 3 d post-injury. **(A–C)** PCA, t-SNE, and hierarchical clustering analyses of transcriptomic profiles showing clear separation between the CRE and NLRP3-CKO groups across Sham, 1 d, 3 d, and 7 d post-TBI time points. **(D–G)** Volcano plots of differentially expressed genes (DEGs) at different time points post-injury.

### 3.3 NLRP3-CKO impacted immune cell polarization on day 1 and attenuated the death of neural cells on day 3 post-TBI

Cell composition changes were investigated using GSVA based on cell-type signature gene sets (M8) from the mouse MSigDB. The results indicated significant alterations in cell-type signatures, primarily on day 1 post-TBI, with the most notable differences observed in macrophages, monocytes, and T-cell aging-related signatures (Supplementary Figures 2A–G). Further exploration of immune cell composition employed the CIBERSORT package (Figure 4A), revealing that, on day 1 post-injury, NLRP3-CKO led to increases in M1 and M2 macrophages, monocytes, naïve CD8^+^ T cells, and Th1 cells while decreasing M0 macrophages, Th17 cells, and activated dendritic cells (DCs) (Figure 4B). By day 7 post-TBI, various T cells, naïve B cells, and resting NK cells were upregulated, whereas immature DC cells and plasma cells were downregulated (Figure 4C). Neural cell composition analysis, based on a deconvolution method, demonstrated that the NLRP3-CKO group exhibited higher proportions of neurons, astrocytes, diverse glial cells, oligodendrocytes, and ependymal cells compared to the CRE group on day 3 post-TBI (Figure 4D). Overall, while the most pronounced transcriptional alterations occurred on day 3 post-injury, fine-tuning modulation of the immune system transpired on day 1, potentially contributing to the dramatic transcriptome changes and neural cell death observed on day 3 post-TBI. Notably, the long-term effects of NLRP3-CKO persisted even on day 7 post-injury.

**Figure 4:**
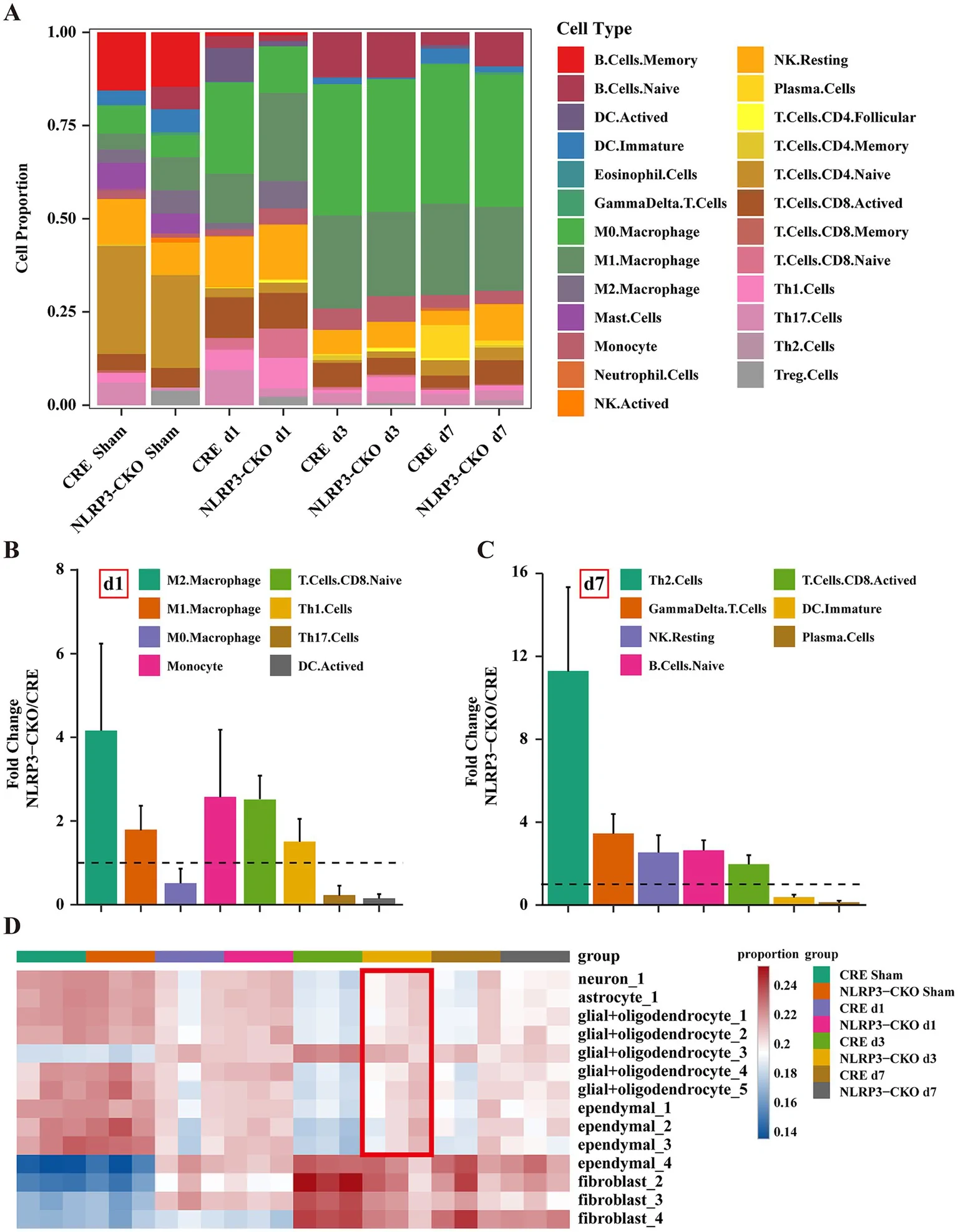
Dynamic changes in immune and neural cell populations at different time points post-TBI. **(A)** Stacked bar plot depicting the proportions of immune cell populations in the CRE and NLRP3-CKO groups at different time points. **(B-C)** Fold change values (NLRP3-CKO/CRE) of immune cell subsets at 1 d and 7 d. **(D)** Heatmap illustrating alteration patterns of neural cell populations in the two groups at different time points.

### 3.4 NLRP3-CKO mitigated the fluctuations of pyroptosis-, neuroinflammation-, and neuropathology-related gene expression post-TBI

To further characterize the effects of NLRP3-CKO on pyroptosis post-TBI, pyroptosis-related genes were screened. Interestingly, NLRP3-CKO mice demonstrated relatively higher levels of pyroptosis-related genes than CRE mice before TBI, but these levels remained largely unchanged post-TBI (Figure 5A). Conversely, in the CRE group, gene levels increased rapidly on day 1 post-injury, peaked on day 3, and remained elevated compared to pre-injury levels on day 7 (Figure 5A). Heatmap analysis highlighted significant differences between the NLRP3-CKO and CRE groups on day 3 post-TBI (Supplementary Figure 3A), with significantly altered gene sets (indicated by the black box) selected for pathway enrichment analysis. KEGG and WikiPathways enrichment analyses revealed that pattern recognition receptor (PRR)-related signaling pathways, cell death pathways, and the IL-1 signaling pathway were downregulated on day 3 (Supplementary Figure 3B). The levels of PRRs (excluding NLRP3) in the NLRP3-CKO group also decreased on day 3 post-injury (Figure 5B).

**Figure 5:**
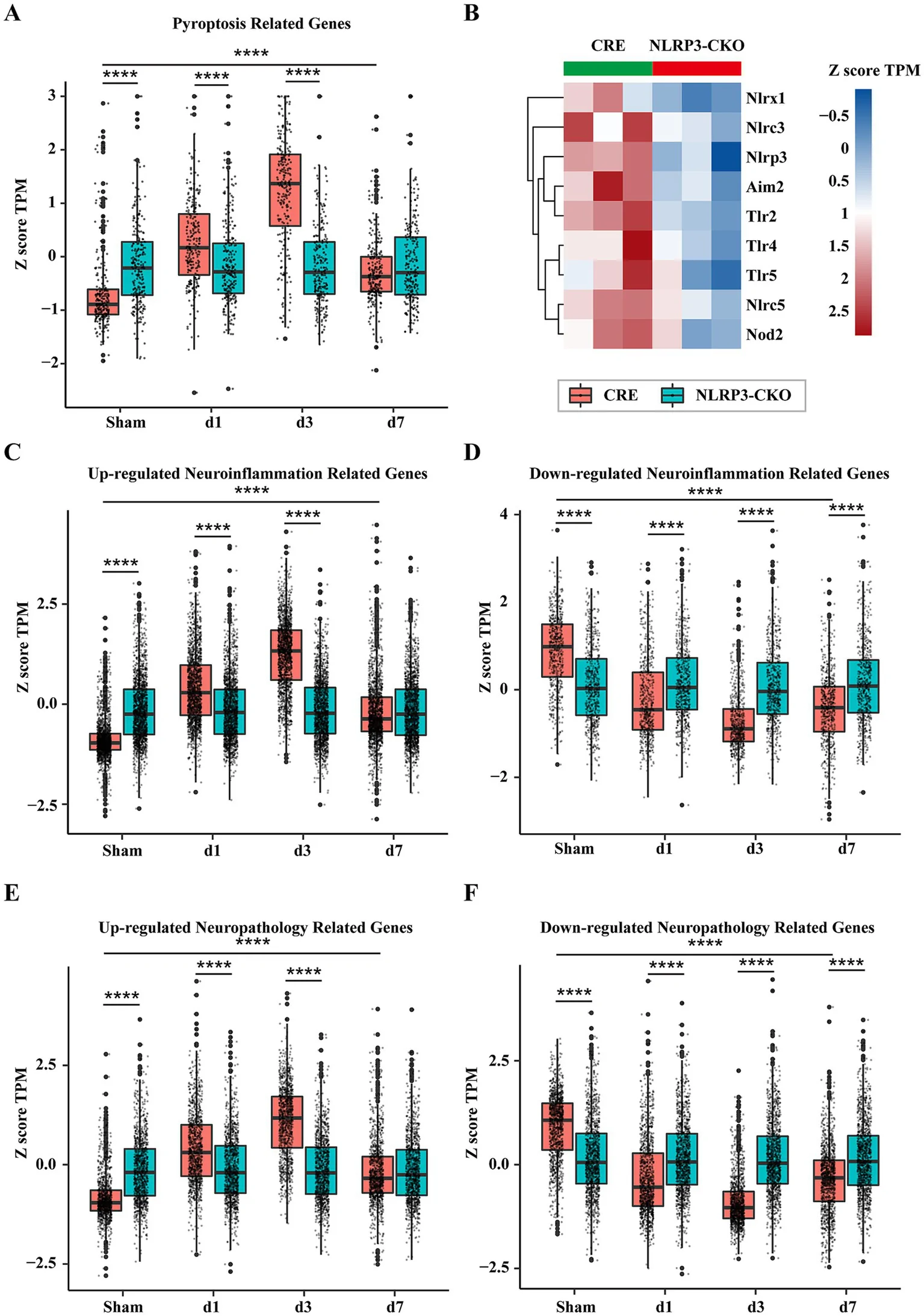
NLRP3-CKO mitigated fluctuations in pyroptosis-, neuroinflammation-, and neuropathology-related gene sets post-TBI. **(A)** Expression levels of pyroptosis-related genes in the CRE and NLRP3-CKO groups across Sham, 1 d, 3 d, and 7 d post-TBI time points. **(B)** Heatmap showing expression levels of diverse PRRs in the CRE and NLRP3-CKO groups. **(C–F)** Box plots presenting z-score-normalized expression levels of neuroinflammation-related **(C,D)** and neuropathology-related **(E,F)** gene sets across time points in the CRE and NLRP3-CKO groups. Upregulated gene sets are shown in panels **(C,E)**, whereas downregulated gene sets are shown in panels **(D,F)**. Gene set expression data were analyzed using a two-factor non-parametric test (Scheirer–Ray–Hare test), followed by Dunn’s *post hoc* test, implemented using the “rstatix” R package.

Dynamic changes in neuroinflammation- and neuropathology-related genes were also assessed. NLRP3-CKO mice exhibited higher levels of upregulated neuroinflammation-related genes compared to CRE mice before TBI; however, these levels remained globally unchanged post-injury (Figure 5C). The NLRP3-CKO group displayed lower levels of downregulated neuroinflammation-related genes prior to TBI, with these levels remaining generally stable post-TBI (Figure 5D). Notably, on day 7 post-injury, the downregulated neuroinflammation-related gene levels in the NLRP3-CKO group were significantly higher than those in CRE mice, contrasting with changes in upregulated neuroinflammation-related genes (Figures 5C,D). Similar dynamic patterns were observed in neuropathology-related genes (Figures 5E,F) (Supplementary Figure 4A). These results highlight the critical role of microglial NLRP3 in the initiation of pyroptosis post-TBI and indicate that microglial NLRP3 exerts extensive effects on other PRRs. Moreover, the findings unravel potential regulatory mechanisms by which microglial NLRP3 influences neuroinflammation and neuropathology-related alterations following TBI.

### 3.5 The landscape of GSEA at different time points post-TBI revealed underlying signaling pathways

GSEA utilizing mouse Hallmark, WikiPathways, and KEGG pathway gene sets was conducted to elucidate the landscape of underlying signaling pathways at various time points post-TBI. For Hallmark gene set enrichment, the NLRP3-CKO group exhibited upregulation of interferon responses at baseline, alongside downregulation of KRAS signaling and coagulation pathways on day 1 post-injury. Notably, on day 3 post-injury, several immune- and inflammatory-related signaling pathways, including IL-2 STAT5, IL-6 JAK/STAT3, and TNF-α signaling via NF-κB, were downregulated (Figures 6A–C; Supplementary Figure 5A). WikiPathways gene set enrichment revealed metabolic alterations in NLRP3-CKO mice at baseline and on day 1 post-injury (Figures 6D,E). In addition, on day 3 post-injury, NLRP3-CKO mice demonstrated downregulation of the microglial pathogen phagocytosis pathway and the TYROBP (TYRO protein tyrosine kinase-binding protein) causal network in microglia, both of which have been strongly associated with cognitive deficits (Zhang et al., 2013) (Figure 6F). No pathways were enriched on day 7 post-TBI based on Hallmark and WikiPathways gene sets. GSEA based on KEGG pathway gene sets identified potential pathways related to infection and metabolism on days 1 and 7 post-injury, while immune- and neuronal-related responses were primarily observed on day 3 (Figures 6G,H), highlighting the significance of the day 3 post-injury time point. Further examination of several representative pathways enriched on days 1 and 3 post-TBI aimed to identify the top regulated genes. In the upregulated KRAS signaling pathway, *Mmp10*, *Plek2*, and *Ccl20* emerged as the top three upregulated genes, while *Cd4*, *Rbm47*, and *Gapt* were the top three in the TYROBP causal network (Figures 6I,J). Noteworthy crosstalk was detected among the IL-6 JAK/STAT3, IL-2 STAT5, and TNF-α signaling pathways, with *Reg1*, *Pf4*, *Il18r1*, *Eomes*, *Ctla4*, *Serpinc1*, *Il12b*, *Olr1*, and *Cxcl5* identified as the top three upregulated genes in each pathway, respectively (Figure 6K). These expression changes were validated by qPCR on days 1 and 3 post-TBI (Supplementary Figure 6). These results elucidate the key pathways involved in the regulation of microglial NLRP3 post-TBI and emphasize that day 3 post-injury serves as a critical time point, with significant alterations in neural-related pathways occurring at this juncture.

**Figure 6:**
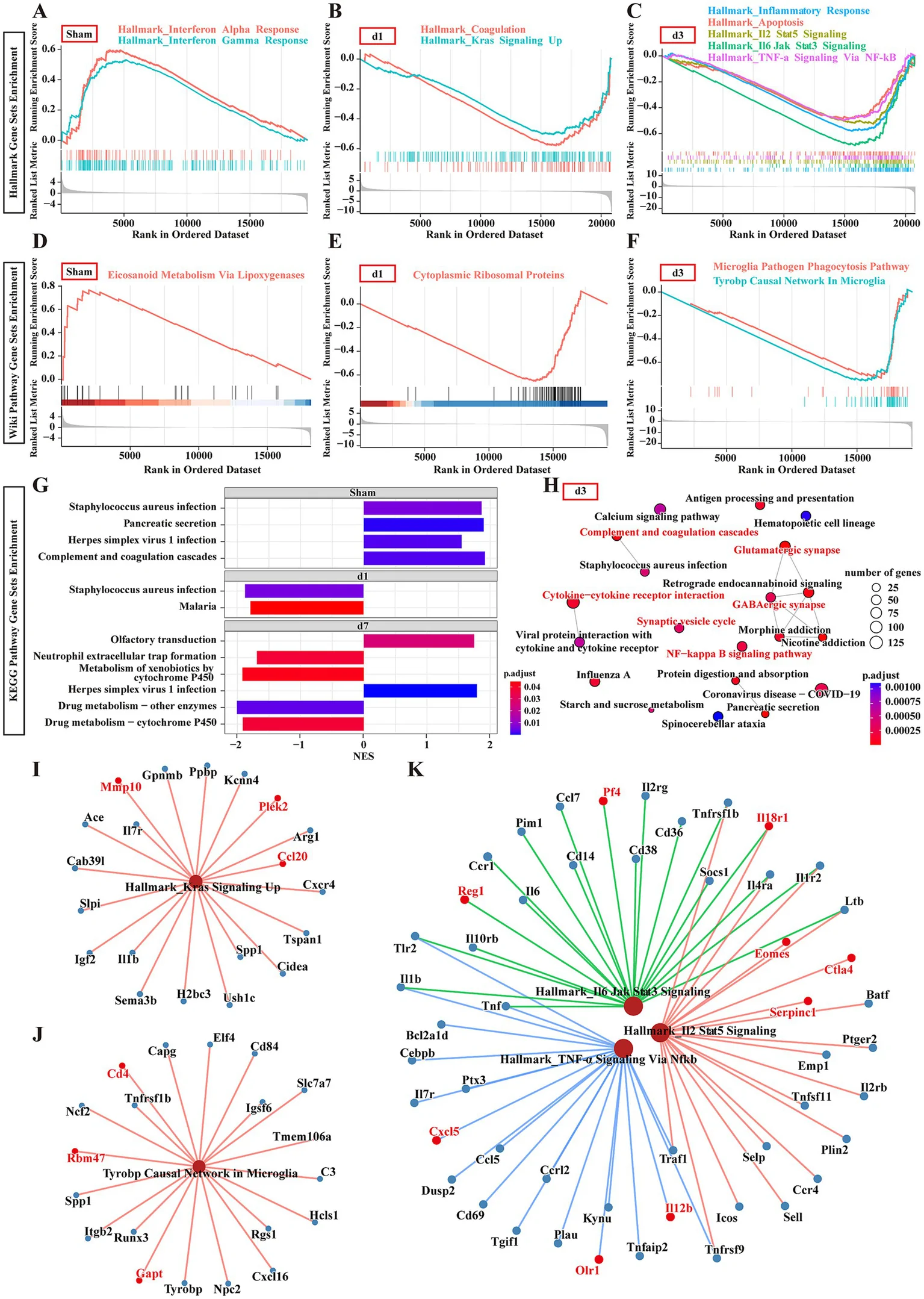
Landscape of the gene set enrichment analysis (GSEA) results at different time points post-TBI. **(A–F)** GSEA plots showing Hallmark **(A–C)** and WikiPathways **(D–F)** enrichment results across Sham **(A,D)**, 1 d **(B,E)**, and 3 d **(C,F)** post-TBI time points. **(G)** Bar plot displaying enriched pathways at 1 d and 7 d post-TBI. **(H)** Network plot visualizing significantly enriched pathways at 3 d. **(I–K)** Core gene networks for Hallmark pathways at 1 d and 3 d post-TBI.

### 3.6 Temporal and pathway enrichment analyses revealed the underlying changes in NLRP3-CKO mice on day 7 post-injury

Temporal analysis utilizing the FCM algorithm was conducted to investigate gene variation trends between the groups. In total, six clusters were characterized in each group based on optimal parameters, and intersection analyses were performed for clusters across both groups (Figures 7A–C). Cluster 4 (C4) in the CRE group and Cluster 3 (C3) in the NLRP3-CKO group shared 850 common genes but exhibited differing expression trends, particularly on day 7 post-injury. Genes in C3 of the NLRP3-CKO group remained downregulated, whereas those in C4 of the CRE group demonstrated an upregulation trend on day 7 post-TBI. In addition, C4 clusters from both groups shared 1,146 common genes, and C6 of the CRE group shared 529 genes with C4 of the NLRP3-CKO group; these gene sets displayed divergent trends (upregulation vs. downregulation) on day 7 post-injury. C3 of the CRE group and C2 of the NLRP3-CKO group shared 818 genes but exhibited distinct change patterns (flat vs. upregulated) on day 7. Overall, the temporal analysis revealed that gene clusters in the two groups displayed diverse alteration trends, particularly on day 7 post-TBI, suggesting that NLRP3-CKO may exert long-term effects on the injury response. Subsequent KEGG pathway enrichment analysis of these clusters was performed to comprehensively understand the change trends across different pathways. Notably, several neurodegeneration- and metabolism-related pathways demonstrated differing trends between the CRE and NLRP3-CKO groups (Supplementary Figures 7A, 8A).

**Figure 7:**
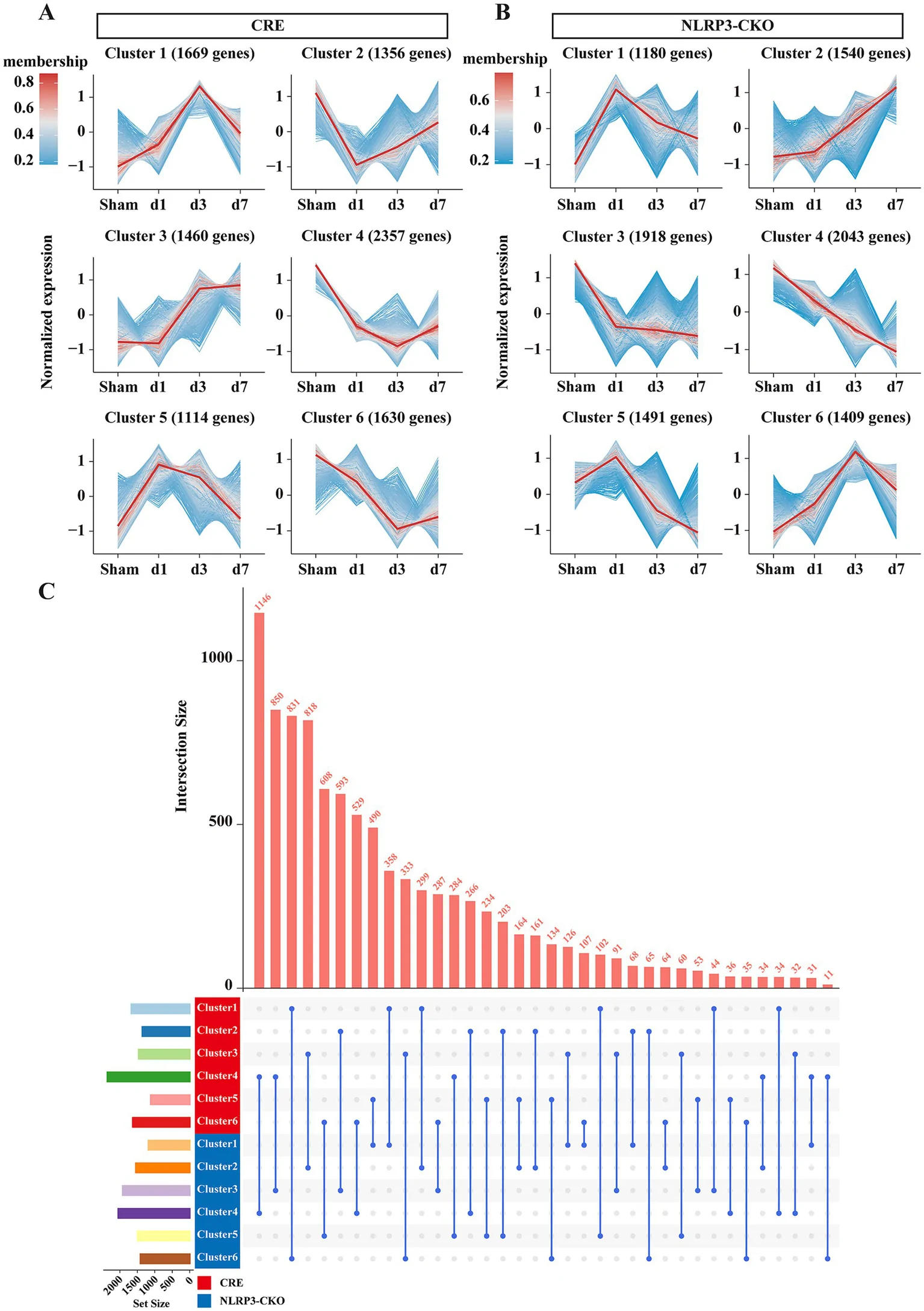
Temporal profiles and intersections of gene clusters generated by the fuzzy c-means algorithm. **(A,B)** Dynamic changes in distinct clusters identified by fuzzy c-means clustering in the CRE and NLRP3-CKO groups. **(C)** Intersection analysis of fuzzy c-means gene clusters between the two genotypes.

Common genes between clusters exhibiting different change patterns were subsequently screened, followed by KEGG pathway enrichment analysis based on these genes. The results revealed significant differences in diverse neural synapse-, neurodegeneration-, and metabolism-related pathways between the CRE and NLRP3-CKO groups (Figures 8A–D). In addition, three specific signaling pathways—the oxytocin, retrograde endocannabinoid, and phospholipase D signaling pathways—were enriched, and the associated genes were presented (Figures 8E,F). Both the oxytocin and retrograde endocannabinoid signaling pathways included several common genes, with *Camk4*, *Plcb4*, and *Gabra1* identified as the top upregulated genes (|fold change| > 1.4) within these pathways (Figure 8E). In the phospholipase D signaling pathway, *Lpar1* and *Plpp3* emerged as the top upregulated and downregulated genes, respectively (|fold change| > 1.4) (Figure 8F). These expression changes were validated by qPCR on day 7 post-TBI (Supplementary Figure 9). Overall, the temporal analysis indicated that subtle changes persisted between the CRE and NLRP3-CKO groups on day 7 post-TBI, with the oxytocin, retrograde endocannabinoid, and phospholipase D signaling pathways playing crucial roles in disease progression at this time point.

**Figure 8:**
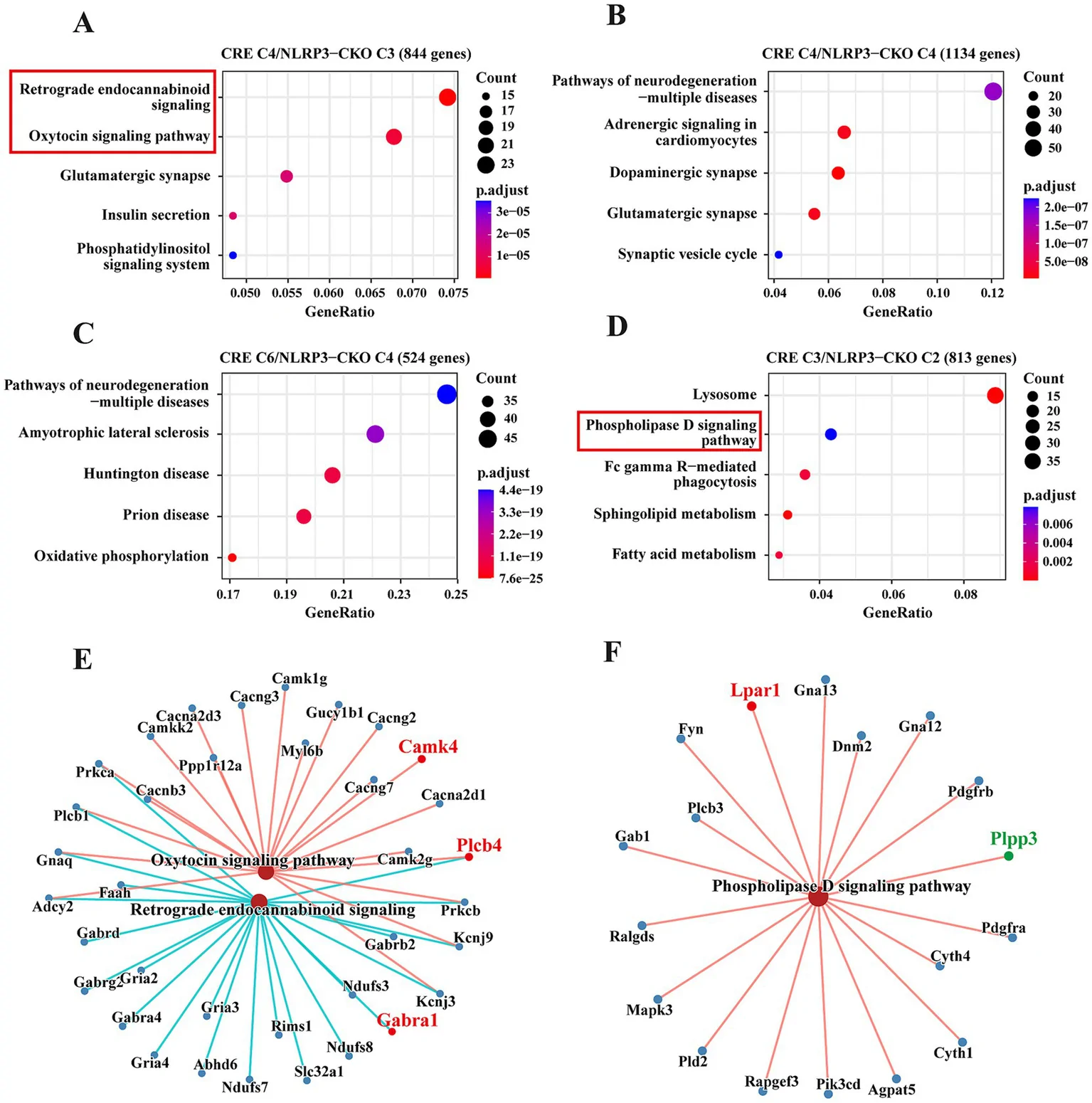
KEGG pathway enrichment analysis of genes within the top intersecting clusters between the CRE and NLRP3-CKO groups. **(A–D)** Bubble scatter plots showing KEGG pathway enrichment for the major intersecting gene clusters. The x-axis represents the gene ratio; dot size corresponds to the number of enriched genes, and dot color reflects the adjusted *p*-value. **(E,F)** Network plots of three enriched signaling pathways. Upregulated genes are labeled in red, while downregulated genes are labeled in green.

## 4 Discussion

In this study, NLRP3-CKO was found to attenuate neurological deficits and neuropathological alterations during the acute phase of TBI. Transcriptomic profiling revealed the dynamic impacts of NLRP3-CKO on immune responses, neural cell states, pyroptosis, neuroinflammation, neuropathology, and their underlying signaling pathways across distinct post-injury time points.

Accumulating evidence links microglial NLRP3 inflammasome activation to exacerbated neuroinflammation and neurodegeneration following TBI, with its genetic or pharmacological inhibition consistently conferring neuroprotection (Zheng et al., 2023; Xu L. et al., 2024; Xu S. et al., 2024; Guo et al., 2026; Jia et al., 2026). However, most previous studies have relied on global knockout models or systemic pharmacological inhibition (Montazeri-Khosh et al., 2025; Cao et al., 2026), which cannot disentangle the cell-type-specific contributions to pathology. To date, only two published studies have utilized cell-type-specific NLRP3-CKO mice—both in spinal cord injury models—to verify NLRP3 functions in astrocytes and microglia separately (Zheng et al., 2023; Song et al., 2025). These studies have focused on individual cell types in spinal cord injury, leaving a gap in the comprehensive understanding of cell-type-specific mechanisms in TBI. To address this, microglial NLRP3-CKO mice were generated in conjunction with a TBI model. Observations suggest that microglial NLRP3 acts as a key upstream regulator of post-TBI neuroinflammation, with its deletion resulting in improved acute neurological outcomes. Complementary research has highlighted the time- and region-dependent transcriptional regulation of the brain NLRP3 inflammasome, emphasizing the importance of investigating its temporal dynamics following brain injury (Ciani et al., 2025).

Neuroinflammation represents a highly dynamic process, and its pathological impact shifts over time after injury. Prior research often relies on static observations at a single time point (Zhou et al., 2017). For instance, investigations of NLRP3 in TBI typically analyze one specific post-injury time point (such as 24 or 72 h) to correlate inflammatory activation with neurological outcomes (Zhou et al., 2017; Cakir-Aktas et al., 2023). Similarly, studies of the HDAC3 inhibitor RGFP966 in TBI focus on the 72-h *in vivo* time point, despite observing benefits at 24 h *in vitro* (Xu L. et al., 2024; Xu S. et al., 2024). To address this limitation, a multi-time point dynamic transcriptomic strategy was adopted to systematically profile NLRP3-dependent changes across the acute and subacute phases of injury. Days 1, 3, and 7 post-TBI were selected as study time points: NLRP3 protein levels peak within 24 h post-TBI (Wallisch et al., 2017); by day 3 post-TBI, other inflammasomes such as NLRP1 and AIM2—whose elevation is restricted to day 1—have already subsided, allowing for the analysis of NLRP3-dominated inflammatory responses (Du et al., 2022); and day 7 falls within the subacute phase, representing a critical window for NLRP3 inflammasome-mediated persistent neuroinflammation (Zhou et al., 2022). The central role of microglial NLRP3 was confirmed, revealing its divergent contributions across pathological stages. This combined cell-specific and temporal approach captures the dynamic nature of microglial NLRP3 signaling and provides preliminary mechanistic insights for staged anti-inflammatory intervention.

Comprehensive pathway analysis between NLRP3-CKO and control mice identified key signaling pathways modulating activity at different time points, with temporal trajectory analysis revealing potential long-term shifts in pathway dynamics. Distinct pathways at various time points orchestrate disease progression by modulating NLRP3 inflammasome-driven neuroinflammation and neuropathology.

On days 1 and 3 post-injury, significant downregulation of the KRAS signaling pathway, the TYROBP causal network in microglia, and interleukin-/TNF-α-related pathways was observed in NLRP3-CKO mice. As an upstream regulator of inflammatory activation, KRAS signaling drives microglial polarization and pro-inflammatory transcriptional programs via the mitogen-activated protein kinase (MAPK) cascade during the early injury phase (Wofford et al., 2019; Park et al., 2026). TYROBP, a core myeloid adaptor protein, sustains pro-inflammatory microglial phenotypes and perpetuates inflammatory responses (Zhou et al., 2024). Downstream cytokines such as IL-6 and TNF-α further amplify inflammatory cascades through positive feedback loops, directly triggering neuronal apoptosis and compromising the blood–brain barrier (Crowley et al., 2017). The reduced expression of these inflammatory signature genes in NLRP3-CKO mice suggests that microglial NLRP3 deficiency may suppress early inflammatory transcriptional responses following TBI. By dampening KRAS-driven early inflammatory priming, TYROBP-mediated microglial overactivation, and cytokine-mediated inflammatory injury, NLRP3 deletion mitigates secondary damage to neurons and the blood–brain barrier, offering protective effects against acute TBI.

Temporal profile analysis identified the enrichment of the oxytocin, retrograde endocannabinoid, and phospholipase D signaling pathways on day 7, which exhibited distinct long-term expression patterns. At this time point, microglial oxytocin and retrograde endocannabinoid signaling were significantly downregulated in NLRP3-CKO mice, while microglial phospholipase D signaling was markedly upregulated. Oxytocin signaling functions as an upstream neuroprotective cascade, exerting anti-inflammatory effects and preserving GABAergic synaptic integrity during the subacute post-injury phase (Runyan et al., 2021; Chen et al., 2023). Retrograde endocannabinoid signaling serves as a critical modulator of synaptic homeostasis by restraining excessive glutamate release and maintaining the excitation–inhibition balance, thereby alleviating excitotoxic damage and supporting synaptic remodeling (Fernández-Espejo and Núñez-Domínguez, 2022; Eyolfson et al., 2025). Phospholipase D signaling acts as a central hub for phospholipid metabolism and membrane remodeling, reversing trauma-induced lipid metabolic disorders and providing structural support for neural repair (Sarkar and Lipinski, 2024). These mechanisms may elucidate the long-term effects of microglial NLRP3 on TBI disease progression.

Collectively, our characterization of NLRP3-dependent transcriptional shifts reveals microglial NLRP3-driven inflammatory signatures following TBI and offers preliminary insights for timing-specific anti-inflammatory strategies. Future pharmacological and dual-sex validation studies are necessary to translate these preclinical observations into safer, precision-targeted therapies that limit excessive neuroinflammation and mitigate secondary brain damage in patients with TBI.

## 5 Limitations

Several key limitations should be acknowledged. This study focused on the acute phase of TBI; therefore, investigation of additional early and late time points for more comprehensive kinetic and long-term profiling will be pursued in future research. Bulk RNA sequencing produces averaged signals from heterogeneous brain tissues and cannot resolve cell-type-specific transcriptional changes. In addition, since only male mice were utilized, the generalizability of these results to female mice remains uncertain.

## Data Availability

The datasets presented in this study can be found in online repositories. The names of the repository/repositories and accession number(s) can be found in the article/Supplementary material.

## Footnotes

1
https://github.com/jstjohn/SeqPrep

2
https://github.com/najoshi/sickle

3
https://github.com/kevinblighe/EnhancedVolcano

4
www.gsea-msigdb.org/gsea/msigdb

## Supplementary material

SUPPLEMENTARY FIGURE 1 Transcriptional alterations relative to the Sham group at different time points post-TBI. **(A–C)** Scatter plots showing log_2_ fold change values of genes (relative to the corresponding Sham groups) in the CRE and NLRP3-CKO groups at different time points. Red and blue dots indicate significantly upregulated and downregulated genes, respectively, in both groups.

SUPPLEMENTARY FIGURE 2 NLRP3-CKO diminished signatures associated with macrophages, monocytes, and T-cell senescence, primarily at 1 d post-TBI, according to gene set variation analysis (GSVA). **(A–D)** Heatmaps of cell-type-signature GSVA results in the CRE and NLRP3-CKO groups at different time points. **(E–G)** Heatmaps of macrophage-, monocyte-, and T-cell-related signatures extracted from the red-boxed region in panel **(B)**.

SUPPLEMENTARY FIGURE 3 Expression patterns and pathway enrichment analysis of pyroptosis-related genes. **(A)** Heatmap displaying gene expression profiles across all experimental groups at different time points. **(B,C)** Network plots of differentially expressed pyroptosis-related genes at 3 d [black box in **(A)**], showing significantly enriched pathways identified by KEGG and WikiPathways analyses.

SUPPLEMENTARY FIGURE 4 KEGG and WikiPathways enrichment analysis of neuroinflammation- and neuropathology-related genes. Pathway enrichment analyses were performed on upregulated and downregulated genes related to neuroinflammation (top two rows) and neuropathology (bottom two rows). The left panels show the KEGG pathway enrichment results, and the right panels show the WikiPathways enrichment results.

SUPPLEMENTARY FIGURE 5 Hallmark and WikiPathways gene set enrichment analysis at 3 d post-TBI. **(A,B)** GSEA results based on Hallmark and WikiPathways gene set collections at 3 d post-TBI.

SUPPLEMENTARY FIGURE 6 qPCR analysis of candidate gene mRNA expression at different time points post-injury between the CRE and NLRP3-CKO groups. **(A)** Relative mRNA expression of *Mmp10*, *Plek2*, and *Ccl20* in the Sham and 1 d post-injury groups. mRNA levels were normalized to *Gapdh*. **(B)** Relative mRNA expression of *Cd4*, *Rbm47*, *Gapt*, *Reg1*, *Pf4*, *Il18r1*, *Eomes*, *Ctla4*, *Serpinc1*, *Il12b*, *Olr1*, and *Cxcl5* in the Sham and 3 d post-injury groups. mRNA levels were normalized to *Gapdh*. Quantitative real-time PCR data were analyzed using the unpaired two-tailed Student’s *t*-test. ns, not significant; **p* < 0.05.

SUPPLEMENTARY FIGURE 7 Heatmap and KEGG pathway enrichment analysis of gene clusters in the CRE group. Left: Heatmap illustrating gene expression patterns for individual gene clusters identified in the CRE group. Right: Top enriched KEGG pathways for each cluster. Gene expression values are shown across multiple post-injury time points (Sham, day 1, day 3, and day 7).

SUPPLEMENTARY FIGURE 8 Heatmap and KEGG pathway enrichment analysis of gene clusters in the NLRP3-CKO group. Left: Heatmap illustrating gene expression patterns for individual gene clusters identified in the NLRP3-CKO group. Right: Top enriched KEGG pathways for each cluster across different time points (one-way ANOVA followed by Tukey’s multiple comparisons test among three groups, **p* < 0.05).

SUPPLEMENTARY FIGURE 9 qPCR analysis of candidate gene mRNA expression at 7 d post-injury between the CRE and NLRP3-CKO groups. **(A)** Relative mRNA levels of *Plpp3*, *Lpar1*, *Gabra1*, *Plcb4*, and *Camk4* in the Sham and 7 d post-injury groups. Gene expression was normalized to *Gapdh*. Quantitative real-time PCR data were analyzed using the unpaired two-tailed Student’s *t*-test. **p* < 0.05, ***p* < 0.01.
